# Mycobacterium-Induced Reactive Arthritis in an Older Adult With Rheumatoid Arthritis

**DOI:** 10.7759/cureus.43057

**Published:** 2023-08-07

**Authors:** Mao Yuasa, Kazuki Suyama, Kazuya Adachi, Shiho Amano, Chiaki Sano, Ryuichi Ohta

**Affiliations:** 1 Family Medicine, Shimane University Faculty of Medicine, Izumo, JPN; 2 Community Care, Unnan City Hospital, Unnan, JPN; 3 Community Medicine Management, Shimane University Faculty of Medicine, Izumo, JPN

**Keywords:** rheumatoid arthritis, japan, nontuberculous mycobacteria, poncet’s disease, rural hospital, general medicine

## Abstract

The use of immunosuppressive medications to treat rheumatoid arthritis may trigger the activation of latent mycobacteria, leading to infection. These infections can lead to reactive arthritis. Conversely, both reactive and rheumatoid arthritis may be encountered in the geriatric population. When such complications arise, the treatment process becomes more complicated, necessitating careful consideration of elaborate therapeutic approaches. An 83-year-old man presented to our hospital with subacute back pain and arthralgia of the extremities. The patient was diagnosed with rheumatoid arthritis combined with mycobacterial arthritis. We approached the treatment cautiously by concurrently managing tuberculosis and non-tuberculous mycobacteria (NTM), and administering methotrexate and prednisolone for rheumatoid arthritis. This treatment resulted in remission of both conditions. When treating arthritis in older adults, it is important to consider the possibility of reactive arthritis secondary to mycobacterial infection and rule out latent tuberculosis. Moreover, when rheumatoid arthritis is complicated by mycobacterial infection and during the management of rheumatoid arthritis, the possibility of arthritis exacerbation due to mycobacteria should be considered. Hence, in situations where there is a likelihood of extrapulmonary lesions stemming from Mycobacterium infection, a proactive treatment approach targeting both Mycobacterium spp. and rheumatoid arthritis is indispensable.

## Introduction

Antimycobacterial reactive arthritis is an infrequent yet significant disease when examining arthritis in older adults. Tuberculosis-associated arthritis, caused by Mycobacterium tuberculosis, includes tuberculous arthritis (infection of the joints via the bloodstream) and Poncet's disease (a variant of reactive arthritis that involves an immune response). Tuberculous arthritis occurs in approximately 1%-3% of all tuberculosis cases and is the second most prevalent form of osteoarticular tuberculosis after tuberculous spondylitis [1-3]. Poncet's disease, referring to tuberculosis-related reactive arthritis, is an uncommon condition observed across various arthritic manifestations, from minimal arthritis to polyarthritis, with a notable predilection for the ankle joints. The disease duration varies between three days and six years [4]. Although the number of cases is limited, reports have suggested an association between the disease and extrapulmonary lesions. Additionally, sacroiliac arthritis is less common than the other types of reactive arthritis. In most cases, anti-tuberculosis therapy leads to remission, and the involvement of HLAB27 and DQB1*0301 alleles has been postulated [4,5]. Non-tuberculous mycobacteria (NTM) can also induce reactive arthritis, primarily through direct pathogen transmission from environmental sources or adjacent infected foci, such as surgical procedures, trauma, or needle injection [6]. Reports on reactive arthritis caused by NTM are limited.

The use of immunosuppressive drugs for rheumatic diseases increases the risk of mycobacterial infection, and consequently, reactive arthritis. Patients with rheumatoid arthritis have approximately twice the risk of tuberculosis and six times the risk of complications from NTM compared with individuals without underlying health issues [1]. Additionally, Mycobacterium spp. can cause pulmonary and extrapulmonary infections, with arthritis being recognized as one of the infection manifestations [2]. Moreover, the use of immunosuppressive drugs further elevates susceptibility to reactive arthritis caused by tuberculosis and NTM [1]. We report the case of an 83-year-old man who presented to our hospital with a chief complaint of subacute back pain and arthralgia in the extremities. He was diagnosed with rheumatoid and antimicrobial arthritis. Herein, we discuss the differentiation and treatment of rheumatoid arthritis and mycobacterial arthritis.

## Case presentation

An 83-year-old man presented to our hospital with a chief complaint of back pain. He was originally independent in activities of daily living (ADL). One year before his visit, he was treated with prednisolone by his primary physician for suspected polymyalgia rheumatica. Approximately 15 days before the visit, the patient experienced back pain. The pain increased on the day of his visit, and he could not walk, resulting in emergency transportation to the hospital. His medical history included bilateral knee osteoarthritis, hypertension, and angina pectoris. His medications included prednisolone, acetaminophen, carvedilol, furosemide, and nicorandil. The patient had never experienced tuberculosis infection.

**Figure 1:**
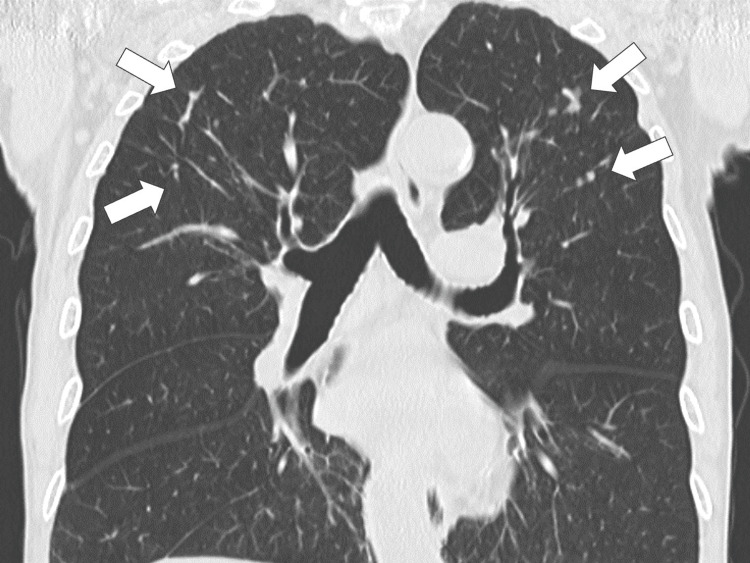
The chest computed tomography revealing predominant nodules in both upper lung fields (white arrows)

Vital signs at the time of admission were: height: 170 cm, weight: 56.1 kg, temperature: 37.0°C, pulse rate: 77/min, blood pressure: 150/80 mmHg, respiratory rate: 18/min, SpO2: 99% (ambient air). Physical examination revealed tenderness in the bilateral shoulder joints, metacarpophalangeal and proximal interphalangeal (MP/PIP) joints of the hands, lumbar region, and both knee joints. He had back pain and fever. Plain computed tomography (CT) of the chest and abdomen was performed to exclude aortic dissection and pancreatitis. The CT revealed predominant nodules in both the upper lung fields (Figure 1).

**Figure 2:**
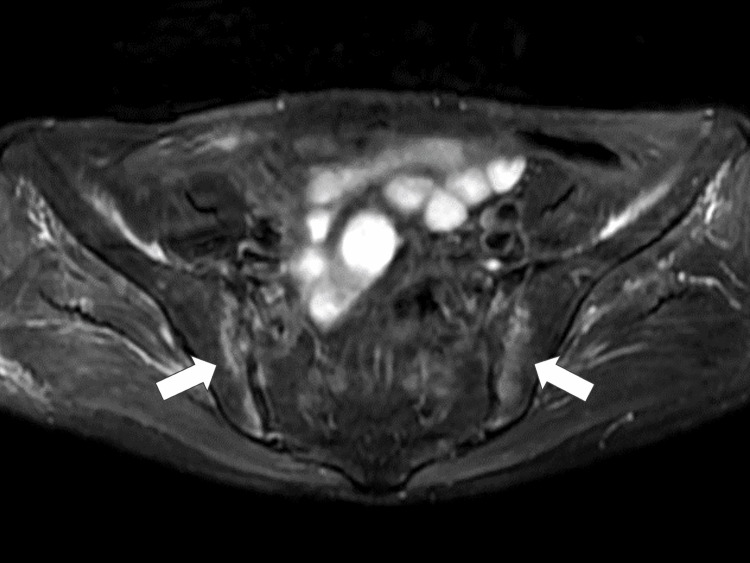
Pelvic magnetic resonance imaging scan revealing high signal intensity on short-tau inversion recovery in the bilateral sacroiliac joints (white arrows)

After admission, three specimens were collected to rule out active tuberculosis. On the third day of hospitalization, the patient experienced tenderness in the shoulder, hands, MP/PIP, and knee joints. Blood tests indicated an inflammatory response, with a blood sedimentation rate of 13 mm, C-reactive protein (CRP) level of 8.64 mg/dL, rheumatoid factor of 456 IU/mL, and anti-cyclic citrullinated peptide (anti-CCP) antibody level of 491 U/mL. Rheumatoid arthritis was considered a possible diagnosis; however, further investigation was required because of the inability to definitively rule out other conditions. On the fifth day of hospitalization, pelvic magnetic resonance imaging (MRI) revealed high signal intensity on short-tau inversion recovery (STIR) in both sacroiliac joints, and the possibility of sacroiliac arthritis was considered (Figure 2).

The elevated levels of rheumatoid factor, antinuclear antibodies, and anti-CCP antibodies, coupled with the tendency for symptoms to be exacerbated under long-term prednisolone therapy, suggested the possibility of a systemic chronic inflammatory disease. Additionally, the initial T-SPOT (an interferon-gamma release assay) was inconclusive, prompting the consideration of latent and disseminated tuberculosis infection.

On the ninth day of hospitalization, he experienced tenderness in both sacroiliac joints and fluid retention in both hands and knee joints. Loop-mediated isothermal amplification (LAMP) and tuberculosis culture of joint fluid from both knees were negative. We conducted the T-SPOT (second time) and quantiferon (one of the interferon-gamma release assays); the T-SPOT was non-judgemental, and the quantiferon was negative. On the 20th day of hospitalization, gastric fluid LAMP and culture for tuberculosis were performed, which were negative for gastric fluid LAMP, but positive for gastric fluid culture. Considering the presence of pulmonary involvement, generalized joint symptoms, atypical sacroiliac arthritis symptoms, and a high risk of tuberculosis activation due to immunosuppression, the patient was clinically diagnosed with Poncet's disease or tuberculous arthritis. On the 29th day of hospitalization, four-drug combination therapy (rifampicin, isoniazid, ethambutol, and pyrazinamide) was initiated as an antituberculosis treatment. On the 32nd day of hospitalization, considering the coexistence of rheumatoid arthritis as a complication, methotrexate 4 mg was initiated in addition to the continued administration of prednisolone (3 mg). However, owing to pronounced arthritic symptoms, the prednisolone dosage was increased to 10 mg on the 33rd day of hospitalization. On the 42nd day of hospitalization, the prednisolone dose was further increased to 25 mg, resulting in a discernible improvement in peripheral joint pain. Although the pain persisted in both the knee and right wrist joints, the knee joint ameliorated following the administration of triamcinolone acetonide via injection.

On the 44th day of hospitalization, the patient was diagnosed with Mycobacterium avium subsp. avium, and blood tests were positive for Mycobacterium avium complex (MAC) antibodies. On the 46th day of hospitalization, clarithromycin was initiated as a therapeutic intervention for sacroiliac arthritis caused by extrapulmonary NTM. On the 54th day of hospitalization, the patient experienced only residual pain in the right wrist, which improved. Therefore, the patient was placed in follow-up care without additional treatment. The number of inflamed joints decreased from 14 (on the 41st day) to one (on the 50th day), and the pain in the right sacroiliac joint was completely resolved. The patient showed favorable progress and was transferred to the rehabilitation ward.

## Discussion

In this case, distinguishing between rheumatoid arthritis and mycobacterial reactive arthritis was challenging, thus, emphasizing the importance of enhancing a patient’s quality of life through appropriate empirical therapy, symptom monitoring, and treatment reinforcement for autoimmune arthritis.

When dealing with polyarthritis in older adult patients, differentiating between rheumatoid arthritis and mycobacterial infection, including mycobacterial reactive arthritis, remains challenging and requires consideration of various differential diagnoses. Rheumatoid arthritis typically manifests as a symmetrical erosive disease that affects multiple joints. Although it can affect any joint, it is most commonly observed in the MP/PIP, and metatarsophalangeal joints, wrists, and knees. The involvement of the distal interphalangeal, sacroiliac, and lumbar joints is less common [7]. In contrast, tuberculous arthritis commonly presents as monoarthritis in weight-bearing joints, such as the hips or knees [2]. Unlike bacterial arthritis, tuberculous arthritis progresses slowly [3]. However, multifocal involvement has also been reported [8]. Given its chronic course, initial synovitis progresses to periarticular bone loss, marginal erosion, and eventual joint destruction; the clinical manifestations of tuberculous arthritis can sometimes resemble those of rheumatoid arthritis, posing challenges in differentiation [6,9,10]. The gold standard test for distinguishing between rheumatoid and tuberculous arthritis is the detection of Mycobacterium tuberculosis through joint fluid culture; however, the positive rate of this test ranges from 20% to 40% [11]. Tuberculous arthritis affects the sacroiliac joints, as observed in this case, and accounts for 5%-10% of skeletal tuberculosis [12]. A biopsy of the joint or synovial membrane serves as the definitive diagnostic method; however, in this case, a biopsy was not performed because of the patient’s risk-benefit profile [12].

Reactive arthritis should also be considered when treating resistant polyarthritis and rheumatoid arthritis in older individuals. Poncet’s disease, a type of reactive arthritis associated with tuberculosis, is a possible diagnosis. The diagnostic criteria for Poncet's disease are not uniform. Even the criteria proposed by Sharma et al. rely on excluding other diseases and responses to tuberculosis treatment [4,13]. Therefore, the diagnosis of Poncet's disease remains unclear. The fact that sacroiliitis is unlikely to occur is inconsistent with this case and cannot be ruled out as a potential differential diagnosis [4]. In the present case, NTM was detected in the gastric fluid culture, suggesting the possible involvement of NTM. It has also been reported to increase the risk of NTM infection. However, whether this association is attributable to the pathogenesis of rheumatoid arthritis or the immunosuppressive drugs used for its treatment has not been adequately investigated [1,14]. Although rare, NTM can also infect the musculoskeletal system, often through direct transmission from environmental sources or adjacent infected foci, such as surgical procedures, trauma, or needle injections [6]. Rheumatoid arthritis complicated by NTM arthritis has also been reported [15]. Therefore, the possibility of NTM involvement in sacroiliac arthritis cannot be excluded from the differential diagnosis. Cases of pulmonary NTM infection complicated by polyarthritis have also been reported, suggesting the possibility of reactive arthritis caused by NTM [16].

Enhancing the treatment of autoimmune arthritis in older patients is essential to improve their quality of life. Standard therapeutic modalities for rheumatoid arthritis include conventional synthetic disease-modifying antirheumatic drugs (csDMARDs), molecularly targeted synthetic disease-modifying antirheumatic drugs (tsDMARDs), biological disease-modifying antirheumatic drugs (bDMARDs), nonsteroidal anti-inflammatory drugs, and corticosteroids [17]. Certain medications exert immunosuppressive effects, so their use should be carefully considered in patients with concurrent Mycobacterium tuberculosis infection. Complications arising from Mycobacterium tuberculosis infection pose a challenge. Diverse viewpoints exist on this matter. The medical guidelines issued by the Japanese Respiratory Society suggest administering methotrexate at the attending physician's discretion, advocating the use of corticosteroids, and generally discontinuing the use of biological agents [16].

Conversely, an emerging consensus favors the use of biological agents. Supporting this notion, studies have reported that the administration of high-dose prednisolone and etanercept substantially expedites the response to tuberculosis treatment [14]. In the context of NTM, no evidence suggests an association between steroids or methotrexate and a worsened prognosis. Therefore, the decision rests solely with the attending physician. Although biological agents are generally contraindicated, their use is permissible under specific circumstances, such as in the presence of MAC or radiographic lesions indicative of nodular or bronchiectasis, after a meticulous assessment of the risk-benefit ratio. Several cases involving patients with rheumatoid arthritis with pulmonary MAC complications who continued receiving biologics and appropriate anti-NTM therapy have been reported [14]. In this case, the patient was undergoing treatment with methotrexate and corticosteroids. However, we are considering using biological agents to evaluate the treatment efficacy. Notably, the referenced guidelines and reports exclusively pertain to cases complicated by pulmonary infections, and there is inadequate evidence for cases complicated by extrapulmonary infections. Therefore, considering the patient's specific condition, biologics should be used with the utmost caution.

## Conclusions

We report a case of rheumatoid arthritis complicated by mycobacterial sacroiliitis. Given the rarity of sacroiliac arthritis in rheumatoid arthritis cases and the presence of laboratory findings indicative of mycobacterial infection, we considered mycobacterial arthritis as a potential complicating factor for reactive arthritis resulting from mycobacterial infection. It is crucial to consider the likelihood of arthritic complications arising from mycobacterial infections when managing rheumatoid arthritis and during treatment. Achieving an optimal balance of the immune system becomes challenging when mycobacterial infections complicate rheumatoid arthritis, leading to diverse perspectives on treatment approaches. Moreover, the evidence regarding the management of extrapulmonary complications is inadequate. Herein, we report a case of rheumatoid arthritis in a patient receiving meticulous treatment with methotrexate and prednisolone, which substantially improved despite concurrent mycobacterial infection. We demonstrated the feasibility of implementing an assertive treatment approach for rheumatoid arthritis, even in patients with extrapulmonary NTM.
